# Biological alterations in renal and hepatic tissues by a novel gold (III) anti-cancerous compound 

**DOI:** 10.22038/IJBMS.2018.28622.6935

**Published:** 2018-10

**Authors:** Ayesha Ahmed, Abdulaziz M Mansour Alkhawajah, Dalal M Al-Tamimi, Mohamed A Shawarby, Anvarhusein A Isab, Ahmed Badar

**Affiliations:** 1 Department of Pathology, College of Medicine, Imam Abdulrahman Bin Faisal University and King Fahd Hospital of the University, Dammam, Saudi Arabia; 2 Department of Pharmacology, College of Medicine, Imam Abdulrahman Bin Faisal University, Dammam, Saudi Arabia; 3 Department of Chemistry, King Fahd University of Petroleum & Minerals, Dhahran, Saudi Arabia; 4 Department of Physiology, College of Medicine, Imam Abdulrahman Bin Faisal University, Dammam, Saudi Arabia

**Keywords:** Anticancerous, Kidney, Liver, Novel gold III compound, Toxicity

## Abstract

**Objective(s):**

Newer organo-metallic, specifically gold (III) complexes with multiple ligands are currently being formulated with primary focus of having increased anti-cancerous properties and decreased cytotoxicity. In this study, histological toxicity profile of a newly formulated anti-cancerous gold (III) compound [trans-(±)-1,2-(DACH)_2_Au]Cl_3_ Bis(trans-1,2-Diaminocyclohexane) was investigated by evaluation of kidney and liver tissues of rats treated by the compound.

**Materials and Methods:**

This is a quasi-experimental study. In acute toxicity component of the study, (n = 16) male rats weighing between 200–250 g were administered single, variable concentration of the gold (III) compound, [trans-(±)-1,2-(DACH)_2_Au]Cl_3_ Bis(trans-1,2-Diaminocyclohexane) to determine LD_50_ (dose that is lethal to 50% of rats). An IP injection of 2.3 mg/kg (equivalent to 1/10 of LD_50_) was injected for 14 consecutive days to (n=10) male rats in the sub-acute component of the study. Autopsy preservation of liver and kidney tissue in buffered formalin, sample processing, histopathological evaluation, and comparison with unremarkable controls (n=5) was conducted sequentially.

**Results:**

A dose of 2.3 mg/kg did not produce any tubular necrosis in kidney specimens. Mild interstitial inflammation with prominence of plasma cells was the main histological alteration. Plasmacytic pyelitis was also seen. Varying extents of cytoplasmic vacuolization and mild focal lobular and portal inflammation were predominant hepatic microscopic findings.

**Conclusion:**

[trans-(±)-1,2-(DACH)_2_Au]Cl_3_ Bis(trans-1,2-Diaminocyclohexane) produced no histological damage in renal and hepatic tissues of rats. This very limited sample animal-based study points to the relative safety of this new gold compound. However, there is a need to compare this compound with established drugs in a comparative non-animal based study.

## Introduction

Cisplatin or *cis*-diamminedichloroplatinum II with its strong anticancerous properties was discovered in the mid-1960s. It was the first platinum-based anti-cancerous drug that was formulated for clinical use. This started the search for newer platinum-based anticancerous compounds (1). It is clinically utilized in adults for treating solid tumors in the lungs, stomach, colon, breast, uterus, cervix, urinary bladder, CNS, prostate, testis, and ovaries, etc. It is also employed in children in hematological tumors (2). Limitations of prolonged cisplatin administration include many toxic manifestations like bone marrow suppression, gastrotoxicity, hepatoxicity, ototoxicity, and allergic reactions. Renal toxicity is, however, the major dose-limiting side effect of cisplatin, which commonly presents as acute kidney injury in approximately 20-30 % of patients besides other presentations of renal damage (2). Additionally, drug resistance is also a major factor that limits drug utilization. These serious adverse factors led to the development of newer platinum-based compounds like picoplatin, satraplatin, and the multinuclear platinum complex BBR3464 (triplatin). These drugs in clinical trials did not prove to be very effective due to their deficient diffusion capability in solid tumors (3). 

The current medicinal chemistry and drug designing hence targets towards formulation of new metal-based compounds with different pharmacokinetics from platinum-based drugs, with an aim to develop a compound with increased anticancerous potential and limited side effects. In this regards, gold (III) complexes have currently acquired great importance due to their anti-cancer cell proliferative ability by mechanisms entirely different from the cisplatin modus operandi along with showing less adverse side effects (3). 

The structural and electronic profiles of gold (III) compounds bear similarity to platinum (II) compounds (4). Gold (III) being isoelectronic and isostructural to platinum became the potential candidate for investigations and research as a new anticancerous compound. Gold (III) compounds were initially abandoned for many years as they had the drawback of being sensitive to light and relatively unstable, with easy reducibility to metallic gold under physiological conditions. Later on, in order to overcome these drawbacks, gold (III) compounds with various ligands like cyclam, polyamines, phenanthroline, and terpyridine, etc. were formulated. These compounds were extensively investigated by various spectroscopic modalities and were attributed a satisfactory stability profile. This was followed by extensive *in vitro* pharmacological analysis and also *in vitro* testing of cancer cell lines, which provided promising results (5) 

The actual breakthrough of gold compounds utilization is not just for the treatment of cancer, but to design them in a way that they are clinically more potent and effective with less toxicity and potential selectivity towards cancer cells. Their potential mode of action must rely on their delivery and improved uptake in cancer-specific cells thus decreasing the possibility of undesirable side effects (1). 

There are only a few documented reports in literature highlighting the toxicity profile of gold (III) anticancerous compounds (6,7). We have already designed a gold (III) compound [Au(en)Cl_2_]Cl (where en = ethylenediamine) with negligible toxicity in liver and kidney of rats as compared to other clinically utilized anticancerous agents (8). 

The current study was a further advancement in exploring new gold III compounds that could be potent against cancer stem lines and would show limited tissue toxicity and damage. In this attempt Au(III) compounds containing various 1,2-Diamminocyclohexane (DACH) isomers, which are (*R,R-1,2-DACH*); (*S,S,-1,2-DACH*); and (*Cis-1,2-DACH*) were investigated. The *in vitro* cytotoxicity (IC_50_ values) of these was previously screened against different types of cancer cell lines, and [Au{*trans*-(±)-1,2-(DACH)}_2_]Cl_3 _had the best cytotoxic effects against prostate (PC3) and gastric (SG79901) human cancer cell lines (9). The present* in vivo* study was carried out with an aim to evaluate biological and histopathological alterations in renal and hepatic tissues by the DACH gold (III) compound. In order to assess the extent of damage that could be caused by these compounds to these vital tissues. This compound has already proved to have promising anticancerous potential (9). Evaluation of its renal and hepatic toxicity will be a step further in validating its future clinical potential in the process of new drug development. 

## Materials and Methods

This study was approved by KACST (project no. 14- MED64-04) and conducted at Department of Pathology, King Fahd Hospital of Imam Abdulrahman Bin Faisal University, Dammam, KSA after mandatory approval by IRB of the university**. **This is a quasi-experimental study. The study comprised two experimental groups of animals along with a third control group. The first experimental group was designated as acute toxicity group and the second as subacute toxicity group. For the study, Wistar male rats (n=31) weighing in the range of 200–250 g were obtained from the animal house of the University of Dammam. These were kept in standardized conditions where the temperature was maintained at 23±2 ^°^C under a 12 hr light-dark cycle, humidity 36%, and light intensity of 315 lux 2000. Rats were fed standard chow. Food and water were provided *ad libitum* for one week to acclimatize them before starting the experiment. 

***Acute toxicity study***

The acute toxicity component of the study was further subdivided into 4 groups A-D, with each group comprising 4 animals (total n=16). Each of the group was administered a single dose of the gold (III) chloride compound IP in doses of 20, 30, 40, and 50 mg/kg, respectively. 

After 24 hr the number of dead rats was counted. LD50 (dose that is lethal to 50% of rats) was calculated by a method elaborated by Miller and Tainter (10) which came out to be 23 mg/kg. After 24 hr the remaining alive rats were also sacrificed. For all animals, an autopsy was then carried out. Preservation of kidney and liver tissues of rats was done in 10% buffered formalin. These were then subjected to histopathological analysis. 

***Sub-acute toxicity study***

There were 10 rats in the sub-acute toxicity component of the study. A dose of 2.3 mg/kg (1/10 of LD50) of the gold III compound was injected IP for 14 consecutive days to all animals that were then sacrificed. Autopsy of all rats was followed by preservation of kidney and liver tissues in 10% buffered formalin. The tissues were processed and histopathological evaluation was carried out. 

***Control group***

This group designated as (E) comprised 5 animals. These were given an IP injection of 0.2 ml water for 14 days. All animals were then sacrificed and an autopsy was done with preservation of kidney and liver tissue in 10% buffered formalin. Histopathological analysis of the tissues was then conducted. 

***Tissue processing for histopathological evaluation***

Tissue processing was done in an automated tissue processor (Tissue-Tek VIP-5, from SAKURA). The first step was fixation that consisted of two cycles of immersion of tissue in buffered formalin with each cycle being of two hours followed by removal of fixative by washing the tissue in distilled water for half an hour. Next step was dehydration, which consisted of tissue exposure to an increasing concentration of alcohol (i.e., 70%, 90%, and 100%) for different time durations. The clearing was then done by immersing the tissue for an hour in a mixture of 50% xylene and 50% alcohol and then one and a half hours in pure xylene. Tissue impregnation in molten wax, embedding, paraffin block formation, sectioning (4–5 um) and finally staining with hematoxylin and eosin was done and final slides were prepared for histopathological evaluation (11). 

The slides for renal and hepatic tissues were evaluated using light microscopy and the findings recorded as follows: 

***Histopathological findings in renal specimens***

A grading system documented by Zhang *et. al.* (12), which consists of 5 grades of renal tubular necrosis was adopted. These ranged from normal renal tubules to extensive renal tubular necrosis. The exact grading is as follows:

Grade 0=normal renal tubular histology 

Grade 1=Renal tubular epithelial cell degeneration, with insignificant apoptosis or necrosis.

Grade 2–5=Renal tubular epithelial apoptosis or necrosis comprising <25%, <50%, <75%, and ≥75%, respectively, of the entire renal tubular tissue associated with other tubular and interstitial changes. 

***Histopathological findings in hepatic specimens***

The criterion used by Ramachandran *et al.* (13) was adopted to calibrate the hepatic specimens. The histological alterations seen in drug-induced hepatic lesions were reported as one of the following alterations:

1. Acute hepatitis 

2. Acute liver failure

a. Necrosis with minimal or absent inflammatory response

b. Necrosis with severe inflammatory response

3. Cholestasis

a. Bland cholestasis

b. Cholestatic hepatitis

4. Chronic Hepatitis

5. Granulomatous hepatitis

6. Steatohepatitis

7. Steatosis

a. Microvesicular steatosis

b. Macrovesicular steatosis

c. Mixed micro and macrovesicular steatosis

8. Vascular Abnormalities

a. Sinusoidal obstruction syndrome

• Each lesion was further calibrated as mild, moderate, and severe/focal or diffuse. 

***Statistical analysis***

Chi-square test was used to compare the groups for the significance of difference observed on histopathological evaluation of renal and hepatic tissues. 

## Results

***Acute toxicity study***

*Histological findings in renal tissue*

**Figure 1:**
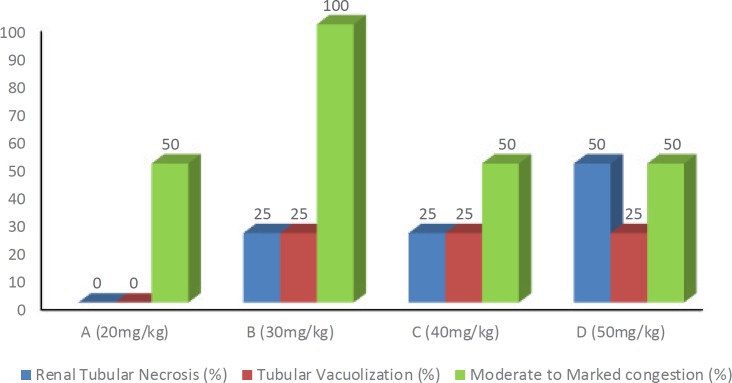
Acute toxicity, overall percentages of renal microscopic findings

The findings in renal tissue consisted of renal tubular necrosis/apoptosis in variable extent (Table 1 and Figure 1). 

In group A (dose: 20 mg/kg) all the four rats revealed normal renal histological findings without any evidence of renal tubular necrosis. Mild to moderate congestion was the only prominent histopathological feature present. 

**Figure 2:**
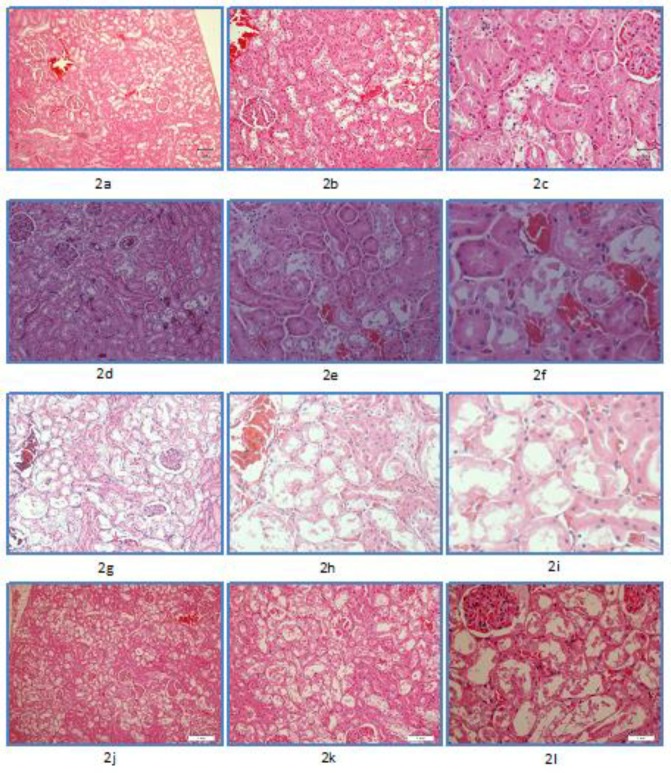
Acute toxicity, renal microscopic findings. Using Hematoxylin and Eosin staining at magnifications of x10, x20, and x40.

In group B (dose: 30 mg/kg), all animals died spontaneously before sacrificing. Renal tubular necrosis of varying range was observed. Two animals revealed grade 1(Figures 2a-2c), one grade 2 (Figures 2d-2f), and one grade 3 tubular (Figures 2g-2i) necrosis. Both animals with grade 1 necrosis also showed varying extents of cytoplasmic vacuolization.

Group C (dose: 40 mg/kg), all animals died spontaneously before sacrificing. Two animals revealed grade 2 renal tubular necrosis and one each grade 1 and grade 3. Focal cytoplasmic vacuolization was also seen.

Group D (dose: 50 mg/kg) again had all animals dead before sacrificing. Two animals revealed grade 4 renal tubular necrosis (Figures 2j-2l) and one animal grade 1. One animal had no renal tubular necrosis. Focal cytoplasmic vacuolization was also evident. 

The variable extent of congestion was also identified in all the groups of acute toxicity study.

There was no renal toxicity observed at the dose of 20 mg/kg (group A). This was significantly better (*P*-value <0.05)as compared to the other three test groups and comparable to the control group. However, there was significant (*P*-value <0.05) moderate to marked congestion observed in group B as compared to other groups and the control (as shown in Table 1).

*Hepatic microscopic findings*

**Figure 3:**
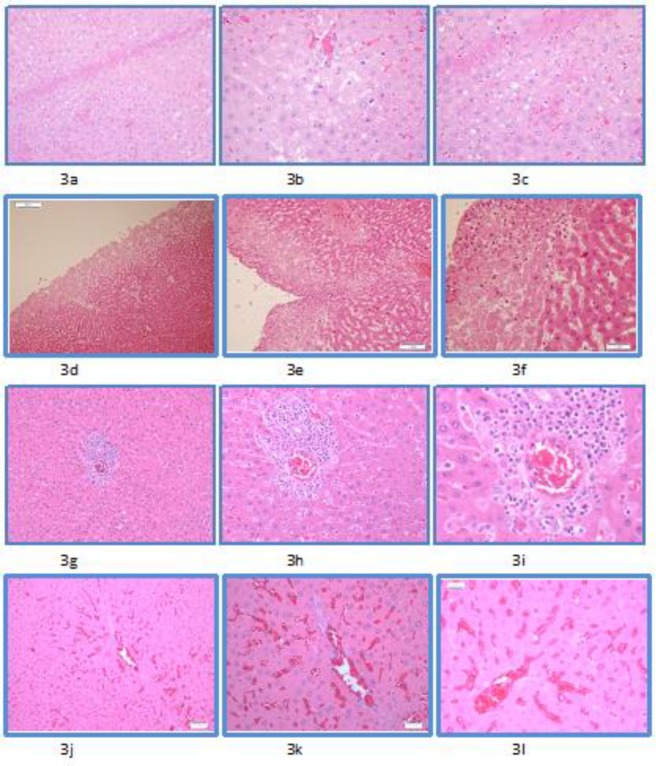
Microscopic picture of hepatic tissue showing steatosis, capsular inflammation, portal inflammation, and cytoplasmic vacuolization as seen in acute toxicity component of the study. Using Hematoxylin and Eosin staining at magnifications of x10, x20 and x40

The hepatic microscopic findings consisted of varying extents of steatosis (Figures 3a-3c), capsular inflammation (Figures 3d-3f), degeneration and necrosis, predominantly sub-capsular, lobular and portal inflammation (Figures 3g-3i), along with congestion 

(Figures 3j-3l). The percentages are depicted in Table 2. Additionally, focal edema was seen in 25% of cases in groups A and C. Necrosis was predominantly seen in the sub-capsular region. 

***Control group***

**Figure 4:**
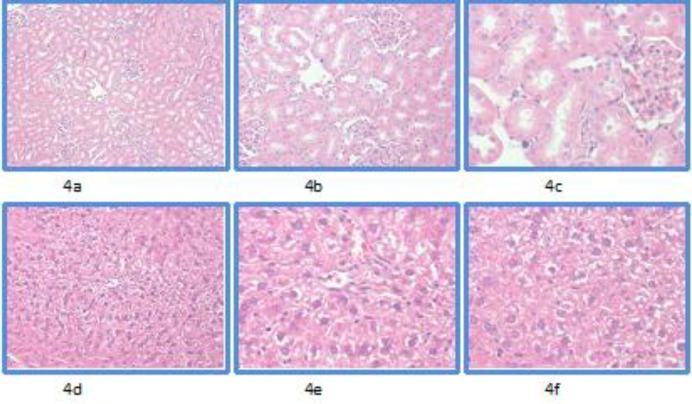
Microscopic findings seen in kidney and liver tissues in the control group. Using Hematoxylin and Eosin staining at magnifications of x10, x20, and x40

The control group (E) revealed unremarkable normal histological findings in renal tubules (Figures 4a-4c).

**Table 1:** Main histological findings in kidney tissue in acute toxicity

**Groups A-D** **Each group having n=4**		**A**	**B**	**C**	**D**	**E (control)** **n=5**
**Dosage mg/kg**		20	30	40	50	0
**Renal tubular necrosis grade 1-5**	**5**	0	0	0	0	0
	**4**	0	0	0	50% (n=2)	0
	**3**	0	25% (n=1)	25% (n=1)	0	0
	**2**	0	25% (n=1)	50% (n=2)	0	0
	**1**	0	50% (n=2)	25% (n=1)	25% (n=1)	0
	**0**	100%* (n=4)	0	0	25% (n-1)	100% (n=5)
**Percentage of interstitial inflammation / Pyelitis**	**None**	100% (n=4)	100% (n=4)	100% (n=4)	75% (n-3)	100% (n=5)
	**Mild**	0	0	0	25 (n=1)	0
	**Moderate to marked**	0	0	0	0	0
**Extent of congestion **	**None**	0	0	0	0	100% (n=5)
	**Mild**	50% (n=2)	0	50% (n=2)	50% (n=2)	0
	**Moderate to marked**	50% (n=2)	100% (n=4)*	50% (n=2)	50% (n=2)	0
**Extent of tubular vacuolization **	**None**	100% (n=4)	75% (n=3)	75% (n=3)	75% (n=3)	100% (n=5)
	**Focal **	0	25% (n=1)	25% (n=1)	25% (n=1)	0
	**Diffuse **	0	0	0	0	0

* : *P-*value <0.05 on *x*^*2*^ test when compared with the controls

**Table 2:** Main histological findings in liver tissue in acute toxicity study

**Groups A-D** **Each group having n=4**		**A**	**B**	**C**	D*	**E (control)** **n=5**
**Doasge mg/kg**		**20**	**30**	**40**	**50**	**0**
**Steatosis**	**None**	0	0	50% (n=2)	50% (n=2)	100% (n=5)
	**Mild**	100%* (n=4)	100%* (n=4)	25% (n=1)	50% (n=2)	0
	**Moderate**	0	0	25% (n=1)	0	0
	**Severe**	0	0	0	0	0
**Hepatocellular degeneration or necrosis**	**None**	100% (n=4	75% (n=3)	100% (n=4	50% (n=2)	100% (n=5)
	**Single cell degeneration**	0	25% (n=1)	0	0	0
	**Necrosis along with inflammation**	0	0	0	50** (n=2)	0
**Portal and lobular inflammation**	**None**	50% (n=2)	75% (n=3)	100%* (n=4)	100% (n=4)	100% (n=5)
	**Mild**	50% (n=2)	25% (n=1)	0	0	0
	**Moderate**	0	0	0	0	0
	**Marked **	0	0	0	0	0
**Extent of congestion **	**None**	50% (n=2)	50% (n=2)	0%	100%* (n=4)	100% (n=5)
	**Mild**	50 (n=2)	50 (n=2)	100 (n=4)	0	0
	**Moderate to marked **	0	0	0	0	0
**Capsular acute inflammation **	**None**	100% (n=4)	75% (n=3)	75% (n=3)	75% (n=3)	100% (n=5)
	**Mild**	0	25% (n=1)	25% (n=1)	25% (n=1)	0
	**Moderate to marked **	0	0	0	0	0

* : *P-*value <0.05 on *x*^*2*^- test when compared with the controls and other groups

(scale bar denotes the percentage of renal tubular necrosis, renal tubular vacuolization, and extent of congestion seen in the renal specimen of animals in groups A-D)

Figures 2a-c: Grade 2 renal tubular necrosis. 2a: x10 2b: x20 2c: x40

Figures 2d-f: Grade 2 renal tubular necrosis. 2d: x10 2e: x20 2f: x40

Figures 2g-2i: Grade 3 renal tubular necrosis. 2a: x10 2b: x20 2c: x40

Figures 2j-k: Grade 4 renal tubular necrosis. 2j: x10 2k: x20 2l: x40

Figures 3a-c: Mild hepatic steatosis. 3a: x10, 3b: x20, 3c: x40

Figures 3d-f: Capsular inflammation and necrosis. 3d: x10, 3e: x20, 3f: x40

Figures 3g-i: Hepatic portal inflammation. 3g: x10, 3h: x20, 3i: x40

Figures 3j-l: Hepatic congestion. 3j: x10, 3k: x20, 3l: x40

Figure 4a-c: Renal control, unremarkable renal tissue. 4a: x10, 4b: x20 4c: x40

Figure 4d-f: Hepatic control, cytoplasmic vacuolization. 4d: x10, 4e: x20, 4f: x40

**Figure 5:**
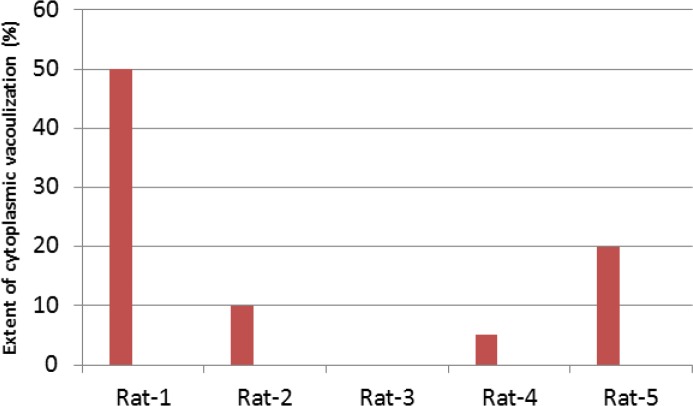
Extent of cytoplasmic vacuolization seen in hepatic tissue of each control specimen

**Figure 6.**
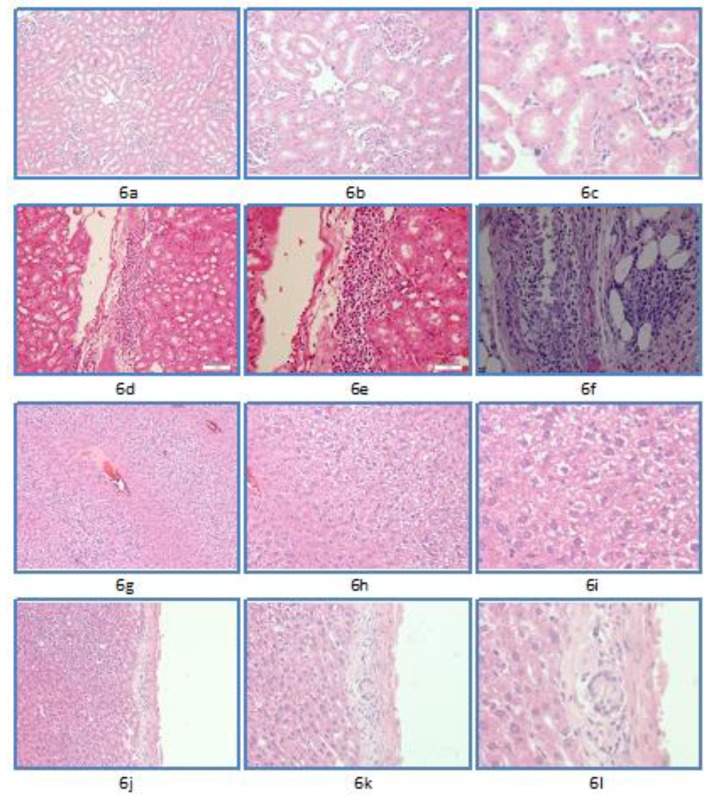
Range of microscopic alteration in kidney and liver tissues as present in the sub-acute toxicity component of the study. Using Hematoxylin and Eosin staining at magnifications of x10, x20, and x40

Figures 6a-c: Unremarkable renal tissue. 6a: x10, 6b: x20, 6c: x40

Figures 6d-f: Plasmacytic pyelitis. 6d: x10, 6e: x20, 6f: x40

Figures 6g-i: Hepatic cytoplasmic vacuolization. 6g: x10, 6h: x20, 6i: x40

Figures 6j-l: Hepatic capsular microgranuloma. 6j: x10, 6k: x20, 6l: x40

The hepatic pathology comprised cytoplasmic vacuolization in variable range (Figures 4d-4f). The extent of cytoplasmic vacuolization seen in each case is presented in Figure 5.

***Sub-acute toxicity***

This batch had a total of 10 animals that were administered a dose of 2.3 mg/kg (1/10 of LD_50_) for two weeks. There was no evidence of renal tubular necrosis in any of the animals of this group. Five (50%) of the animals revealed no histological alteration (Figure 6a-6c). The remaining five (50%) showed occasional foci of interstitial inflammation with prominence of plasma cells with two cases also showing plasmacytic pyelitis (Figures 6d-6f). In the liver, cytoplasmic vacuolization (Figures 6g-6i) was the main finding seen in four (40%) cases, the extent of cytoplasmic vacuolization varied and comprised 30%, 40%, 60%, and 70%, respectively. In addition, four (40%) cases revealed mild focal lobular and portal inflammation with one out of these also showing a portal microgranuloma (Figures 6j-6l). Two (20%) of the cases were unremarkable.

## Discussion

The anticancerous potential of gold DACH compounds has been scarcely investigated as compared to platinum-DACH complexes (14), although these can contribute significantly in this regard.

We demonstrated decreased drug-related toxicity in kidney and liver tissues by a newly formulated gold (III) compound [*trans*-(±)-1,2-(DACH)_2_Au]Cl_3 _*Bis(trans-1,2-Diaminocyclohexane).* In the subacute toxicity part of the study, the rats were subjected to a dose of 2.3 mg/kg of this compound and no evidence of necrosis in renal tubules was identified. The only findings seen were occasional foci of interstitial inflammation with prominence of plasma cells and plasmacytic pyelitis. 

Nephrotoxicity is an intrinsic adverse effect of many anti-neoplastic drugs. The minimum dose mostly needed to significantly reduce burden of the tumor is accompanied by considerable renal damage (15). These cause renal injury by inciting a complex array of renal manifestations such as intra-renal vascular constriction, direct toxic tubular epithelial cell damage, and obstruction of the renal tubules. Additionally renal endogenous potential of drug metabolism can transform a harmless compound into a markedly toxic substance, augmenting the associated damage (16).

We have previously formulated a novel gold (III) compound, [Au(en)Cl_2_]Cl that demonstrated safety for renal tissue in a wide dosage range and was also shown to be safe at a much higher dose levels as compared to other common clinically utilized anticancerous drugs (8) such as cisplatin, 5-fluorouracil, and doxorubicin as other studies have reported. Formulation of the current compound is an effort to further explore the anticancerous potential of gold compounds that bear structural homology and functional capability on par with cisplatin but with markedly diminished nephrotoxicity. 

The compound under discussion showed no renal damage as evidenced by the absence of intra-tubular necrosis at a dose of 2.3 mg/kg in the sub-acute toxicity component of the study. The glomeruli and renal vasculature were also unremarkable. There is a paucity of animal studies evaluating dose wise effects of compounds on the renal tissue so dose wise comparative analysis is hindered. However, a wide range of renal pathologies has been documented with administration of clinically prevalent anticancerous drugs. Carboplatin, a cisplatin derivative, mitomycin C and gemcitabine, which are antitumor antibiotics are known to cause acute kidney injury (AKI) culminating due to thrombotic microangiopathy and presenting as hemolytic-uremic syndrome. The morphological findings comprise fibrin microthrombi in glomerular capillaries and renal arterioles and mucoid material deposition in the intima of arteries and arterioles along with mesangiolysis. In chronic lesions nodular expansion of mesangium, vascular sclerosis, and onion skinning may be observed (17). Cisplatin dose-related nephrotoxicity is caused by its potential to induce apoptosis and necrosis in the cells. This cytological damage may lead to acute tubular injury or tubular necrosis. Cisplatin once inside the cells activates multiple enzymatic pathways involving activation of caspases, protein kinases that can induce mitosis or activate cyclin-dependent kinases, and the p53 tumor suppressor gene signaling system (18). How gold DACH complexes act at sub-cellular levels is yet to be fathomed but many novel gold III complexes with different ligands have been reported to induce an alteration in the balance between pro-apoptotic and antiapoptotic factors with overexpression of Bax, a proapoptotic protein and decreased expression of Bcl-2, an antiapoptotic molecule (19). This different mechanism of action could be the reason for renal tissue preservation observed in our set of cases. 

Interferons used to treat various malignancies are reported to cause minimal change disease associated with podocyte injury, focal segmental glomerulosclerosis, and also AKI (20). Ifosfamide an alkylating agent is employed for certain malignancies, comprising some forms of lymphoma, sarcomas, and testicular cancer. The main side effects of ifosfamide include tubulopathies with histopathology revealing features of necrosis and injury in renal tubular epithelial cells (21). An antifolate agent, pemetrexed impairs synthesis of DNA and RNA by inhibiting enzymes involved in metabolism of pyrimidines and purines in malignant mesothelioma and pulmonary malignancies. It has been documented to cause acute renal tubular necrosis, acute interstitial nephritis, tubular atrophy, and also chronic tubulointerstitial fibrosis (22). A dihydrofolate reductase inhibitor, methotrexate, can cause acute tubular necrosis when used in the treatment of high-grade lymphomas (23).

Lack of renal tubular necrosis was observed in our set of cases in sub-acute toxicity part of the study at a dose of 2.3 mg/kg (8). This dosage value is, however, far less than that of our previously reported drug in which LD50 was 32.2 mg/kg. This limitation needs to be attributed a significant weight. However, in a preceding, already reported part of the same study, the anticancerous potential of this compound was calibrated against different cancer stem lines and it was demonstrated to be strongly potent in very small doses (9). In that part of the study, the time-dependent inhibitory effect by MTT assay was conducted against prostate and gastric cancer cell lines. After treatment with 10 μm of the compound, a considerably high anticancerous potential was noted after 24 and 72 hr of treatment, against both the PC3 prostate cancer cells and gastric cancer cells. There was also concentration dependent* in vitro *cytotoxicity on the proliferation of both cancer cell lines evaluated in terms of their IC_50_ (inhibitory concentration that kills 50% of the cells). This study provides an important baseline for more extensive investigation into the upcoming evolving role of gold (III) complexes against specific cancer cell lines (9). 

The presence of plasma cells in renal tissue is an additional though not a consistent finding. This may mainly be due to an immunological reaction against the compound. Several anticancerous compounds including the classical clinically established chemotherapeutics and agents for targeted therapies are known to initiate tumor-specific immune responses. The immune system in the tumor microenvironment has an impact on tumor progression and response to therapy. The configuration of intratumoral lymphocytes and genetic polymorphisms of immune modulators modify the therapeutic outcome (24). The variation in expression in our experimental animals may be because of the different immunological profile of various animals.

Hepatotoxicity as a side effect of anti-cancerous agents has not been as thoroughly investigated as nephrotoxicity as it is not usually a treatment defining or treatment modifying factor (25). However, concomitant hepatic damage is a frequent accompaniment in cancer chemotherapy. The extent of hepatic damage is however idiosyncratic and unpredictable with severity ranging from mere asymptomatic elevation of hepatic enzymes to acute hepatic failure, or a progressive fibrosis culminating into terminal hepatic disease. The morphological patterns can show a wide spectrum of histological alterations like steatosis, cholestasis, inflammation, or vascular presentation such as hepatic vascular occlusive disease (26). 

In the sub-acute toxicity component of the study, the newly synthesized DACH complex of gold (III) revealed variable extents of cytoplasmic vacuolization, focal lobular and portal inflammation, occasional microgranulomas, capsular inflammation, and congestion. Liver injury is often complex and presents itself as a combination of histopathologic alterations rather than a single, definite, well-defined finding (27). One of the most common and earliest manifestations of drug-induced hepatic injury in humans is intrahepatic cholestasis (28). Cholestasis not only has local effects but can also induce hepatocellular apoptosis and necrosis. This effect is produced by bile acid-mediated activation of mitochondrial pro-apoptotic pathways, Fas and TRAIL (tumor necrosis factor-related apoptosis-inducing ligand) mediated activation of hepatocyte death receptors, oxidative stress, and also by some other mechanisms (29). The drug under consideration did not reveal even this earliest form of hepatic injury in any of the animals in the sub-acute toxicity component of the study. Severe intrahepatic cholestasis can be caused by administration of antimetabolites like mercaptopurine and cytarabine or with immunotherapy drugs like interferon (30). Mercaptopurine can also cause hepatitis, rarely fulminant hepatic failure. Platinum agents like oxaliplatin are associated with hepatic vascular injury including sinusoidal congestion of various degrees, perisinusoidal fibrosis, and fibrotic venular occlusion. Hepatic vascular disease can also be caused by thioguanine, alkylating agents like busulphan, and antitumor antibiotics (AA) like actinomycin and mitomycin. Dacarbazine an AA may cause hepatic necrosis with thrombotic occlusion of the small vessels. 5-Fluorouracil has also been implicated in causing sinusoidal obstruction. Additionally, it may also cause steatosis and steatohepatitis. This alteration may also be caused by topoisomerase inhibitors and platinum agents. Chemical hepatitis and biliary sclerosis can be caused by fluorodeoxyuridine and hepatic fibrosis and cirrhosis by methotrexate. Nodular regenerative hyperplasia is caused by thioguanine and platinum agents like oxaliplatin (30-35). 

In the acute toxicity part of the study, the histopathological findings revealed mainly mild steatosis and capsular inflammation. Necrosis confined predominantly in the sub-capsular region was seen in two animals receiving a dosage of 50 mg/kg and one in the 30 mg/kg group. Many studies have reported hepatoxicity induced by cisplatin (36, 37). In a study by Kart *et al.* cisplatin in a single intraperitoneal dose of 6.5 mg/kg was documented to have caused moderate to marked vacuolar degeneration involving the centrilobular hepatic zones and extending towards the portal areas. In severely affected cases perivenular hepatocytic necrosis was also seen (38). Hydropic and vacuolar degeneration, marked hepatocytic disorganization, sinusoidal congestion, and perivenular fibrosis with expanded portal areas have been reported in another study at a dose of 10 mg/kg of cisplatin (25). Necrosis in our drug was seen at a much higher concentration and that too predominantly confined to the sub-capsular area. 

The findings of lack of renal tubular necrosis and minimal hepatic alterations along with its efficacy already being tested against gastric and prostatic cancer stem lines (9) make this compound a promising candidate for further evaluation and investigations. There is a dire need for development of such drugs that may have the indigenous quality of renal safety, which is a major drawback in most cancer therapies. Our work has its inherent limitations. It is a small scale study based exclusively on morphological alterations seen on routine microscopy. However further research incorporating larger cohorts, hematological investigations depicting underlying renal and hepatic pathology, and immunohistochemical and electron microscopic studies to detect the earliest possible changes not visible by routine microscopy need to be conducted. Additionally, in-depth analysis of the sub-cellular mode of action also needs to be fathomed. The current study just opens up the door for this wide array of investigations that could reap a beneficial reward for cancer patients in dire need of a new renal and hepatic friendly drug. 

Cytoplasmic vacuolization was a histological entity that was also present in the control group (E) rats. This overlapping presentation implies that drug toxicity may not be the only mechanism causing this manifestation in liver tissue. Postmortem hepatocyte vacuolization has been reported in the hepatic tissue of mice, rats, guinea pigs, rabbits, monkeys, guinea pigs, mice, and rats. It starts developing immediately on autopsy when respiration ceases, and progresses markedly if rats remained for prolonged times within anoxic chambers. A postmortem high intrahepatic blood pressure and hepatic anoxia are two major contributory factors. Anoxia increases hepatocytic permeability, and high-pressure forces plasma from the sinusoids into hepatic cells to form the intracytoplasmic vacuolization (39). 

## Conclusion

The compound [*trans*-(±)-1,2-(DACH)_2_Au]Cl_3_ *Bis(trans-1,2-Diaminocyclohexane*) produced no histological damage in the renal and hepatic tissues of rats in the sub-acute toxicity part of the study at a dose of 2.3 mg/kg. This very limited sample animal-based study points to the relative safety of this new gold compound. However, there is a need to compare this compound with established drugs in a comparative non-animal based study.
